# Musical Participation, Resilience, and Locus of Control in Musicians from the Margins

**DOI:** 10.3390/bs16040618

**Published:** 2026-04-21

**Authors:** Beatriz Ilari, Graziela Bortz, Nayana Di Giuseppe Germano, Hugo Cogo-Moreira

**Affiliations:** 1 Department of Music Teaching and Learning, Thornton School of Music, University of Southern California, Los Angeles, CA 90089, USA; 2 Music Department, Arts Institute, State University of São Paulo, São Paulo 01140-070, SP, Brazil; graziela.bortz@unesp.br; 3 Department of Music, Federal University of Santa Maria, Santa Maria, 97105-900, RS, Brazil; nayana.germano@ufsm.br; 4 Department of Education, Østfold University College, 1757 Halden, Norway; hugo.c.moreira@hiof.no

**Keywords:** community music programs, psychological resilience, locus of control, margins, Brazil, musical participation, music education, disadvantaged communities

## Abstract

Recent studies suggest that prolonged participation in formal music programs may be associated with the cultivation of resilience and locus of control (LoC) in music students. Brazilian musicians, who were attending or had attended community-based music programs, and a group of matched, untrained individuals from disadvantaged, urban communities completed the Connor–Davidson Scale of Resilience (RISC), the Craig Locus of Control Scale, and the ABEP 2022—Brazilian Criteria of Economic Classification questionnaire. Results suggested that while musical participation alone was not associated with resilience and LoC scores (model 1), a conditional restriction of the same model (model 2) showed a significant interaction between musical participation, age, and RISC and LoC scores, after controlling for SES. Among musicians, higher age was associated with higher resilience scores and internal LoC. Findings from this exploratory study are discussed in light of the multifaceted nature of community-based music programs, the building blocks of resilience and LoC. We also comment on the potential links between resilience and LoC in relation to musical participation and well-being. Limitations of this study are discussed alongside implications for future research.

## 1. Introduction

An assumption that underlies many community music programs serving youth from disadvantaged urban areas is that sustained participation may result in positive gains for individuals and communities alike. Sustained participation in music programs has been associated with gains in emotion regulation (Gerber et al., 2014), socio-cognitive skills (Boal-Palheiros & Ilari, 2023), self-control and behavioral regulation (Alemán et al., 2017), solidarity and coexistence (Cabedo Mas et al., 2025), social resilience (Ussa-Lopez, 2025), and well-being (Pesata et al., 2022; Yi & Kim, 2023), to name a few. While socioemotional skills and well-being have received considerable attention (e.g., O’Hare et al., 2026; Ilari et al., 2019), empirical evidence supporting the emergence of resilience as a byproduct of participation in community-based music programs remains scant. This is paradoxical given that resilience is part of the lexicon of many community music programs, as seen in their mission statements, advocacy materials, and anecdotes (e.g., *Projecto Ritmos de Resiliencia*, Spain; *Música para Resiliencia*, Chile; see also Peres, 2018). Empirical reports examining resilience in community music programs, particularly quantitative studies, are still hard to come by (Zarobe & Bungay, 2017). This could be due to both the diversity of programs and the complexities inherent in studying resilience.

### 1.1. Resilience and Musical Participation

The concept of resilience emerged in the psychological sciences in the 1980s (Ungar, 2012), being initially employed as a metaphor for the capacity of individuals to thrive following trauma (Livingston et al., 2025). Whereas earlier research conceptualized resilience primarily as an individual trait, this construct is now largely viewed as a skill that can be learned. Resilience is also understood to be both a process and an outcome (Livingston et al., 2025). Current research has further extended the definition of resilience beyond individual levels by focusing on structural, social, and ecological issues. Ungar (2012) defined resilience as “a set of behaviors over time that reflect the interactions between individuals and their environments, in particular the opportunities for personal growth that are available and accessible” (p. 14). As research continues to develop, resilience is increasingly recognized as a complex and multifaceted construct, as well as a key component of mental well-being (Zarobe & Bungay, 2017).

Although resilience has been defined in multiple ways within and across different disciplines (Garcia, 2018), there is a degree of overlap between its core factors (Zarobe & Bungay, 2017). Most definitions of resilience include intrinsic and extrinsic factors. Intrinsic factors are those associated with the self, such as the need to belong, sense of competence, self-efficacy (Bandura, 2006), self-concept, and self-esteem. Extrinsic factors, on the other hand, are those related to contextual and social issues, including positive experiences in community spaces, access to support networks of friends and family, social connectedness, and positive relationships (Zarobe & Bungay, 2017). Specific types of resilience—psychological, physical, emotional, social, spiritual, and academic/educational—have also been identified in the literature. These types of resilience are susceptible to the different experiences of adversity that one encounters (Livingston et al., 2025). Close relationships with parents/legal guardians, for example, have been found to be a protective factor and a predictor of resilience in young people (Edwards et al., 2016). By contrast, disadvantage is known to be one of the main causes of adversity and, consequently, of vulnerability in young people (Livingston et al., 2025).

Educational systems can have a direct impact on young people’s capacity to cultivate resilience and experience well-being. Livingston et al. (2025) highlighted how the neoliberal logic underlying educational reforms towards homogenization and data-driven metrics has been detrimental to young people’s learning, development, and well-being. Neoliberal orientations are often based on assumptions that youth have equal opportunities in educational spaces. The common adoption of deficit-based frameworks (e.g., “at risk”) to refer to students perceived as “less prepared” is especially hurtful to those who stem from the margins (Livingston et al., 2025). This approach marginalizes students and teachers, particularly those from disadvantaged communities and minoritized groups. On that note, Hess (2019) defined resilience as “refusing to accept deficit discourses of minoritized youth, while simultaneously naming and/or challenging unjust conditions” (p. 488). Criticizing the resilience turn in U.S. schools, Hess (2019) noted how resilience education often fails to address systemic oppression by reinforcing discourses of vulnerability while fostering student docility and adaptation to oppressive systems. Some would argue that Hess’ definition is more aligned with a resilience strategy known as *protagonism* (Libório & Ungar, 2009) that is discussed ahead. Yet it is undeniable that the deficit-based orientations criticized by Hess (2019) are also seen in the practices of some community-based music programs (see Ilari & Bortz, contracted). These are likely to impact resilience and well-being in youth from the margins.

To gain an understanding of how psychological resilience operates in the context of community-based music education programs in disadvantaged areas, it is vital to understand the different forms of adversity that participants may experience. The nature (i.e., acute or chronic) and duration of adversity offer some clues into one’s capacity to “bounce back.” Acute adversity usually has a short duration and potentially less impact on human functioning, whereas chronic adversity tends to be more detrimental to individuals and communities alike. Chronic adversity can be further divided into two types: *distal onset* and *proximal onset* (Livingston et al., 2025). With no defined starting point, distal-onset adversity has long-lasting effects, sometimes across the lifespan. Poverty and family violence are examples of distal-onset chronic adversity. Proximal-onset adversity, in turn, has a defined starting point and lingers for some time, having an impact on different aspects of an individual’s life. Wars and natural disasters are some examples.

Children and youth are known to adopt active and passive coping strategies when dealing with adversity. Active coping strategies like asking for help and problem-solving have been linked to emotional regulation, mental health, and academic achievement. By contrast, passive coping strategies like avoidance have been associated with anxiety and disengagement (Livingston et al., 2025). Avoidance is a resilience strategy that is sometimes used by young people who experience chronic poverty, a form of distal-onset adversity (Livingston et al., 2025). Another resilience strategy adopted in this context is protagonism, a protective process identified by Libório and Ungar (2009) in their fieldwork with underserved Brazilian youth. Protagonism consists of “strengthening youth participation in processes of social and political transformation, opening space for the recovery of his [sic.] condition as a subject imbued with rights and citizenship” (Stamato, 2009, p. 3). Libório and Ungar (2009, p. 3) define “protagonic actions” as those that are marked by improved personal behaviors and views of the self, and positive engagement with others. These are directly linked to resilience (Libório & Ungar, 2009, pp. 3–4). Marked by a heightened community and civic engagement, protagonism is often manifested through involvement in cultural and artistic activities, such as community music programs.

In musical contexts, resilience has been associated with different forms of active engagement in and with music (Heck et al., 2025). Earlier studies have focused predominantly on adults (Fancourt et al., 2016; Du et al., 2025), conservatoire students and professional musicians (Arbinaga, 2023; Lam, 2024), and clinical populations (Beer & Birnbaum, 2022). Studies with non-clinical, youth populations are rare. Additionally, most existing studies focus on the building blocks of resilience like emotional regulation, self-esteem, belonging, stress modulation, and social support (e.g., Cabedo Mas et al., 2025; Welch et al., 2014) rather than the construct of resilience itself.

Yet, a handful of studies have examined high-level music training and its relationship to performance anxiety, perfectionism, and musicians’ mental health and well-being. Kegelaers et al. (2020) used questionnaires to uncover the associations between resilience and mental health among Dutch music students and professional musicians. They found resilience to be negatively correlated with depression and anxiety. de Bruin (2025) conducted interviews with Australian students (aged 17–18) who were attending secondary school instrumental music programs. Findings suggested that musical practice and participation can contribute to positive emotions and foster well-being. de Bruin also found that musical participation builds resilience in students through teacher and environmental support, and through the self-efficacy gained from skill acquisition.

Concerning children and young people, Pimentel-Aguilar and Ramírez-González (2024) examined whether music education could promote resilience in children (ages 7–15) from a marginalized, rural community in Mexico that was marked by a lack of opportunities and school dropout. Although some girls eventually dropped out of music instruction due to harassment, bullying and family pressure, the authors found the music program to be empowering to the remaining students. In their discussion, Pimentel-Aguilar and Ramírez-González called out the role of the patriarchy in girls’ music education. They also stressed the need for additional research and for the reframing of educational policies. Using participant observation, interviews, and audiovisuals, Ussa-Lopez (2025) found heightened confidence, self-esteem, and social resilience in adolescents who took part in a choir in an area affected by the armed conflict in Colombia. In Hong Kong, Cheung et al. (2024) investigated the effects of 6 months of group music instruction on resilience, depressive symptoms, self-esteem and quality of life of children (ages 8–12) from low-income families. When compared to waitlist controls, children who received music education showed improvements in all four areas at the end of the program, with the resilience outcome exhibiting a large effect size of 0.80. While each of these studies had its own conceptualizations, methodological approaches, and limitations, as a collective, they hint at possible associations between resilience and musical participation in populations from the margins. More studies are needed to tease out the roles of development and musical participation in the cultivation of resilience in children and youth, and to identify possible predictors.

### 1.2. Locus of Control and Musical Participation

Locus of control can be defined as one’s generalized expectancy or perception of the capacity to control the events of their lives (Horst & Jacovidis, 2018; Nowicki et al., 2018). Like resilience, LoC is often conceptualized in terms of a continuum of internal and external beliefs. Internal LoC is the belief that events are contingent upon one’s abilities and actions. It has been associated with positive behaviors, academic achievement, and well-being (Ng-Knight & Schoon, 2017). Internal LoC has also been described as a protective factor from destructive behaviors and violent environments, being a strong predictor of resilience (Edwards et al., 2016). External LoC is the perception that external factors, such as luck, fate, change or the control of others with more power, determine the events in one’s life (Nießen et al., 2022). External LoC beliefs and orientation have been associated with lower self-control, aggression, anxiety, and lower levels of well-being (Ahlin & Lobo Antunes, 2015). In terms of coping strategies, individuals oriented by an internal LoC may adopt coping strategies such as positive thinking and seeking out help. By contrast, those oriented by an external LoC may utilize avoidance and helplessness to cope with adversity (see Graham et al., 2022). Interestingly, the coping strategies used by those oriented by internal LoC vs. external LoC mirror those utilized by, respectively, more and less resilient individuals (Livingston et al., 2025).

Although LoC is often described as a personality trait, recent research suggests that it is a learned response (Horst & Jacovidis, 2018) and “a form of human capital accumulated through parental involvement and investments in education” (Kesavayuth et al., 2022, p. 1). Some of the mechanisms associated with the cultivation of internal LoC include delayed gratification, responsibility, persistence, information gathering, and resistance to coercion (see Nowicki et al., 2018). LoC beliefs and orientations have also been connected to interpersonal relationships, self-reflection, self-efficacy, professional decisions and performance, physical and mental health, and the capacity to cope with stress (Horst & Jacovidis, 2018; Ulas & Yildirim, 2019). While some of the outcomes listed earlier focus on general LoC—one’s overall approach to explain life events as being controlled internally or externally—others tend to be more context-specific (e.g., work-specific LoC).

LoC is influenced by age and context. Studies conducted in the Global North suggest that LoC beliefs change over the life course. Although it is widely accepted that children’s LoC tend to become more internal over time, changes in LoC during middle childhood (around ages 8–16 years) have been documented. These changes could be due to inter- and intra-individual differences (Nowicki et al., 2018). Still, the general increase in internal LoC beliefs and orientation in childhood and adolescence is consistent with the development of children’s independence and agency, including their understanding that actions have an impact on goal achievement (Benson & Steele, 2007). Internal LoC beliefs are said to stabilize in adulthood, becoming more external in later adulthood (see Benson & Steele, 2007; Nowicki et al., 2018).

The contexts that one navigates in everyday life affect LoC beliefs, orientations, actions, and the perception of its “results” (Ahlin & Lobo Antunes, 2015, p. 1804). A study using nationally representative data from U.S. adolescents found differences in LoC based on urbanicity and country region, with school type, race and religion playing small roles. While adolescents from southern regions displayed more external LoC, those from the northeast had more internal LoC. Regional differences aside, higher SES is another factor that has been linked to internal LoC in young people (Ahlin & Lobo Antunes, 2015). Race, ethnicity and minority/majority status, which are sometimes confounded with SES, are also associated with LoC orientations (Ahlin & Lobo Antunes, 2015). In communities ridden by crime and violence, internal LoC may act as a protective factor. Individuals with more internal LoC have been shown to be more resilient to the effects of crime and violence than their external-LoC-oriented peers in these contexts (Churchill & Smyth, 2022).

LoC beliefs and orientations have also been studied in the context of musical participation. Earlier studies (Asmus, 1985; McCormick & McPherson, 2003) offered insights through examinations of students’ internal and external attributions of success and failure in music learning. Miksza (2006) found no significant associations between LoC and performance achievement in brass players, although their LoC scores correlated with impulsivity. Nogaj (2017), who studied LoC and stress coping styles, found that Polish musicians exhibited more internal LoC than visual artists and controls. Once again, while these studies utilized distinct methodologies, they suggest that participation in formal music learning programs may be associated with the cultivation of internal LoC, a predictor of resilience.

### 1.3. Resilience, LoC, and Musical Participation: A Theory of Change

**Figure 1:**
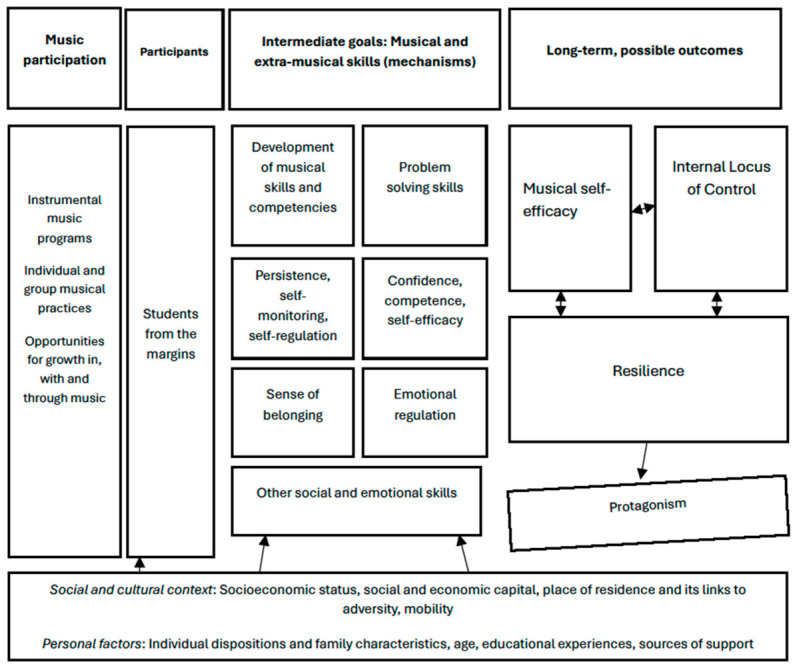
Theory of Change: musical participation, intermediate goals (musical and extramusical skills), and possible long-term outcomes.

The literature reviewed thus far hints at the existence of associations between musical participation, resilience, and LoC. But why would musical participation be associated with resilience and internal LoC? One way to begin addressing this question is through the formulation of a *Theory of Change* that outlines assumptions, mechanisms, and possible steps that could lead to specific goals in an intervention (see Monaghan & King, 2018). While music programs differ in terms of curricula, pedagogical strategies and repertoire, there are some common elements to most of them. Instrumental music programs—like the ones attended by our study participants—often focus on skill development and musical problem-solving. These tasks usually require many hours of dedication through rehearsals, lessons, and individual practice. As students gain musical skills through individual and group practice, they exercise persistence, develop confidence, competence and self-efficacy, exercise executive function skills, and regulate their emotions (Hennessy et al., 2019; Boal-Palheiros & Ilari, 2023; Bortz et al., 2025; Ilari & Cho, 2023; Jiang, 2024). In contexts of distal-onset acute adversity, these skills could potentially act as a buffer against everyday stressors, eventually transferring into feelings of control over one’s immediate environment (i.e., internal LoC) and perhaps into a desire to act (i.e., active strategies of resilience such as protagonism). Figure 1 depicts our conceptual framework, presented here as a Theory of Change.

Our proposed Theory of Change (ToC) departs from the assumption that strong music programs offer opportunities for student growth, in, with and through music. The ToC takes into account the nature of disadvantaged areas and zones of conflict, which are breeding grounds for adversity (Crump et al., 2025). It also considers how individuals from disadvantaged communities often face challenges in many aspects of their lives, including access to education, culture, and leisure. These multiple setbacks are related to different forms of adversity (Livingston et al., 2025) that may foster student perceptions of a lack of control over their lives and their desire to act upon them. Students in communities marked by adversity are also more prone to employ avoidance as a coping strategy (Livingston et al., 2025) instead of protagonism. Therefore, the intermediate goals of music programs in our ToC include the cultivation of musical and extramusical skills such as persistence, belonging, confidence, and a heightened sense of self. These skills, in turn, could potentially strengthen student musical self-efficacy, internal LoC and resilience (including protagonism, as discussed by Libório & Ungar, 2009). Our ToC is a theoretical model that still needs to be tested and refined. This study is a first attempt.

### 1.4. The Current Study: Context, Definitions, and Limitations

This exploratory study draws from a large, multi-methods research project entitled “Musicking the margins.” The research project interrogated the journeys of adult Brazilian musicians from marginal areas, who attended free, socio-musical programs or public music schools in São Paulo and Rio de Janeiro and were admitted to competitive public universities (Ilari & Bortz, contracted). This is a population that is very difficult to access and is not well represented in the literature. Our multi-methods project started in 2021, with data being collected between 2021 and 2022, through in-depth interviews (qualitative component), questionnaires, and scales measuring resilience and locus of control (for quantitative data, see “Methods”). Based on our ToC (Figure 1), this manuscript addresses the quantitative component of the research project. Qualitative data representing the bulk of the research project are presented elsewhere (see Ilari & Bortz, contracted).

In this study, musical participation was defined in relationship to current (i.e., adolescents) or past (i.e., adults) enrollment in formal music instruction for at least one calendar year. It is important to note that musical participation by means of formal instruction in communities marked by poverty is often susceptible to social, economic, and political issues. The latter is known to affect program funding and offerings in different communities (see Sloboda et al., 2020). Additionally, participation in music classes and lessons in disadvantaged communities may become intermittent due to socioeconomic challenges affecting individual students and their families (e.g., students pausing their music education to work and contribute to family income). This was also true during the time of our data collection, when communities were still heavily affected by the Coronavirus pandemic. For these reasons, musical participation was treated in a binary way in this study, being used to distinguish between musicians and untrained individuals. Because adult musicians in this study started their formal music instruction in middle childhood or adolescence, age and enrollment in music programs were taken as a proxy for musical experience.

Another limitation of our study, which was known from the onset, was the bias in our subsample of adult musicians, who had completed several years of formal music instruction. These musicians, who were purposefully selected for the interviews, had attended socio-musical programs in their communities in childhood and/or adolescence, and completed undergraduate degrees (*n* = 29) in music or were nearing completion (*n* = 3). While this was a non-random sample, we understood this to be an opportunity to gather data from a population that is very difficult to reach, offering insights for future research.

### 1.5. Study Aim, Research Questions, and Hypotheses

The aim of this study was to examine self-reports of resilience and locus of control in Brazilian participants (adolescents and adults) from underserved urban communities of São Paulo and Rio de Janeiro, with and without formal music education. Two interrelated questions guided our study:

1. What effect (if any) does participation in music programs and age have on resilience and locus of control scores, after controlling for SES?
2. Is the effect of participation in music programs on resilience and locus of control dependent on age?

Based on the existing literature, we hypothesized that (1) there would be developmental effects (i.e., adults exhibiting higher resilience and more internal locus of control than adolescents); and (2) music participation would influence participants’ responses (i.e., musicians exhibiting higher resilience scores and more internal locus of control).

## 2. Materials and Methods

### 2.1. Sample

Brazilian individuals (*N* = 115) were invited to participate in this study. Among them, there were 66 musicians/music students, 44 matched, non-trained individuals, and 5 participants who did not supply this information. Young music students (adolescents, *n* = 34) were attending a well-known socio-musical program in São Paulo that offers music education through choirs, orchestras and other ensembles for children aged 5–19 from underserved communities (see Bortz et al., 2025). They were recruited through flyers and vocal announcements at their socio-musical program, and their musical participation ranged from 1 to 8 years. As noted, adult musicians (*n* = 32; 16 from Rio de Janeiro and 16 from São Paulo) had attended community music programs and taken part in the qualitative portion of the study. Most adult musicians had recently completed undergraduate music degrees at public universities (or were nearing completion) and ranged between 10 and 25 years of participation in formal music instruction (see Ilari & Bortz, contracted). Adult musicians were recruited through flyers and snowball techniques, with one participant sharing contact information of other potential participants. Matched, non-trained individuals were recruited from the same communities as the musicians from São Paulo, through flyers and vocal announcements at their local schools and community centers. The final sample included 104 participants (age range = 13–52 years; mean = 21.8; SD = 8.7), all from underserved areas located at the margins of both cities.

### 2.2. Data Collection: Instruments and Procedures

All study participants were invited to complete three standardized scales of socioeconomic background, resilience, and locus of control that have been previously validated in Brazil. These instruments with strong psychometric properties, which are readily available in Brazilian Portuguese, were used with all necessary permissions.

- The Connor–Davidson CD-RISC (Connor & Davidson, 2003) is a self-report scale with 25 items, each rated on a 5-point scale, with higher scores reflecting greater resilience. The CD-RISC evaluates resilience across five key areas, namely, (1) personal competence; (2) tolerance of negative affect; (3) positive acceptance of change; (4) sense of control; and (5) spiritual influence and meaning. Scale items include “I am not easily discouraged by failure” and “I am able to handle unpleasant or painful feelings like sadness, fear, and stress.”
- The Locus of Control of Behavior scale (Craig et al., 1984) is a self-report instrument with 17 items measuring one’s perception of control over one’s own behaviors and actions, each rated on a 6-point scale. Higher scores indicate external LoC, whereas lower scores signal internal LoC. Scale items include “I can anticipate difficulties and take action to avoid them” and “People are victims of circumstance beyond their control.”
- The questionnaire of the Brazilian Association of Research Companies—ABEP (2022) is an economic classification criterion created to assess socioeconomic status based on household samples. It contains 15 items that evaluate: (1) ownership of durable consumer goods; (2) type of water supply system and street paving; (3) number of domestic workers employed in the household; and (4) educational level of the head of the family. The questionnaire results in a stratified measure divided into 6 socioeconomic classes: A, B (also subdivided into B1 and B2), C (subdivided into C1 and C2), and DE (see Table 1):

After signing consent forms, adult participants completed the scales and the ABEP questionnaire online, using mobile phones or computers. Adolescent participants assented to participate in the study and completed the scales and the questionnaire using a paper-and-pencil approach at their local schools or community music programs. It was not possible to use online data collection tools in these locations. Earlier studies (e.g., Lefever et al., 2007; Weigold et al., 2013) suggested that online and paper-and-pencil survey data tend to be equivalent. Data collection took anywhere from 10 to 30 min. All data were scored in accordance with the manuals of each scale or questionnaire.

### 2.3. Data Analysis

To conduct the statistical analyses, we used Mplus version 8.0 (Muthén & Muthén, 2017). All data were analyzed using Path Analysis, a special case of Structural Equation Modeling (SEM), employing linear regression under the maximum likelihood estimator. Musical participation was treated as a binary variable. All outcome variables (e.g., Locus of Control of Behavior and Connor–Davidson CD-RISC scores, hereinafter LoC and RISC) were treated as continuous scales, and missing data were treated using full information maximum likelihood under the assumption of a missing-at-random mechanism. The reported results were standardized estimates of STDYX (Standardized Y by X) coefficients, which provide full standardization of both predictors and outcomes.

Our central research question was as follows: *What effect does participation in music programs and age have on RISC and LoC scores*, *after controlling for SES?* Participation in music programs and age were entered as independent predictors, adjusted for SES (i.e., the ABEP variable). We also tested a conditional restriction of the same model where the underlying question was the following: *Is the effect of participation in music programs on RISC and LoC dependent on age?*

The adopted significance level for all analyses was 0.05.

## 3. Results

### Demographics

The total number of participants was 115. Table 2 displays descriptive data for all studied variables. Missing data (see Table 2) were handled using full information maximum likelihood (FIML) estimation implemented in Mplus software, resulting in a final analytical sample of 104 participants aged between 13 and 52 years old (mean = 21.89).

In terms of educational attainment, which is one factor in socioeconomic standing, about 10% of our sample had parents who did not complete elementary education (mothers, *n* = 11; fathers, *n* = 10). Fifteen fathers (14.4%) and 25 mothers (24%) had higher-education degrees, and among this group, only three fathers were declared as heads of the family. In the entire sample, 51 mothers (60%) were declared heads of the household, and several families were led by “solo” mothers. The average SES of the sample (mean = 31.29) corresponded to category B2 of the ABEP questionnaire, with an average monthly income of approximately $1070 (US dollars in 2022).

First, we addressed the question, “*What effect (if any) does participation in music programs and age have on resilience and LoC scores, after controlling for SES?*” The first model showed that participation in music programs had no effect on either resilience or LoC scores. As shown in Table 3, a small and negative correlation was observed between resilience and LoC (r = −0.118), in that higher resilience scores were associated with lower LoC scores, which signals internal LoC. Similarly, increased age was associated with higher resilience scores.

**Figure 2:**
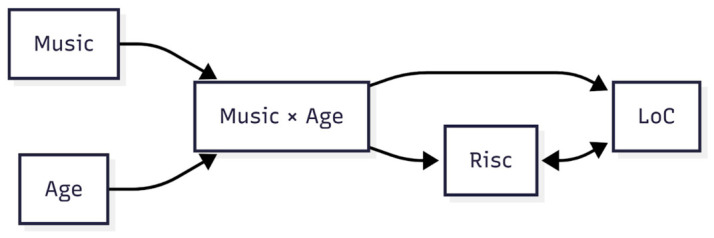
Interaction model in which music participation and age affect resilience and locus of control.

Next, we tested a conditional restriction of the same model to address the question, “*Is the effect of participation in music programs on resilience and LoC dependent on age?*” The results presented in Table 4 show different effects of musical participation depending on age. Among the participants who studied music, older age was associated with higher resilience scores and lower scores for LoC (i.e., more internal LoC). The predictor variables (age and participation in music programs) were not centered and do not affect the model in terms of the size and/or directions of the effects. In our model (Figure 2), age acts as a continuous variable that models itself throughout the development of the sample. This suggests that resilience and internal LoC became stronger over the years for those who participated in music programs.

## 4. Discussion

The aim of this exploratory study was to examine the associations between musical participation and age on resilience and LoC in musicians and non-trained individuals from disadvantaged areas of Brazil. This is a population that has not been well documented in the literature, despite being the main recipient of community music programs.

When analyzed separately and adjusted for SES, there were no effects of musical participation on resilience and LoC (model 1). While musical participation alone did not appear to predict resilience or LoC scores, age was found to be a significant predictor of scores on these two important capacities. This finding is not completely surprising and is consistent with the literature (Benson & Steele, 2007; Livingston et al., 2025; Nowicki et al., 2018). However, both resilience and internal LoC scores appeared to have been affected by musical participation. A second model (model 2), considering the interaction between resilience, LoC, age, and musical participation, showed that in musicians, greater ages were associated with higher resilience scores and lower LoC scores (i.e., more internal LoC). These findings hint at the idea that musical participation could be associated with individuals’ generalized expectancy of their own capacity to cope with and be in control of everyday situations and adversities. Other studies have found a similar, inverse correlation between resilience and internal LoC (Felicia et al., 2022; Horst & Jacovidis, 2018).

Our findings align with earlier studies that found age, along with sustained participation in music programs, to be associated with students’ resilience (e.g., Pimentel-Aguilar & Ramírez-González, 2024; Ussa-Lopez, 2025; Cheung et al., 2024) and internal LoC (Nogaj, 2017). The fact that young music students (i.e., adolescents) but not matched controls (i.e., untrained adolescents) displayed higher resilience and more internal LoC hints at a possible developmental effect (e.g., Benson & Steele, 2007; Nowicki et al., 2018) through music, although this needs to be further substantiated. This finding also lends some initial support to our proposed Theory of Change (Figure 1). Given that our sample was composed of individuals who had experienced distal-onset adversity (Livingston et al., 2025) throughout their lives, our modest findings should be replicated under more controlled conditions, as they have implications for educational and clinical practice.

As noted earlier, a main limitation of our study was the fact that one subsample (i.e., adult musicians) was purposefully selected. For this reason, we recommend caution when interpreting and generalizing our findings beyond the studied participants. Based on our data, we cannot state that resilience and internal LoC were outcomes of prolonged participation in music programs during our participants’ formative years. That is, we cannot rule out that these musicians might have gone through some sort of self-selection process over the course of their music studies (Ilari & Bortz, contracted). It is possible that the adult musicians from our sample remained in their music programs for prolonged periods of time because they already displayed some of the building blocks of resilience (i.e., confidence, self-efficacy and musical self-efficacy, self-control and perhaps even protagonism; see Libório & Ungar, 2009) and/or LoC (i.e., delayed gratification, coercion resistance and responsibility; for persistence, see also Nogaj, 2017). Although beyond the scope of this study, it was noteworthy how our interview data (Ilari & Bortz, contracted) offered hints that the building blocks of psychological resilience and internal LoC were likely operating early in the lives of some but not all adult musicians. As an example, one interviewee described multiple attempts over almost a decade to pass the difficult public university entrance exam (i.e., persistence), and there were multiple commentaries about remaining focused on their studies as future musical and professional “rewards” would eventually emerge (i.e., delayed gratification and developing competencies) (see Ilari & Bortz, contracted). These anecdotes reinforce the need to replicate this study in a more controlled fashion, and possibly by combining quantitative and qualitative data.

It is important to stress that our study took place at a time when the world was still experiencing the disruptions caused by the Coronavirus pandemic. Underserved and marginalized communities were disproportionately affected by COVID-19. If computing precise times of musical participation by means of formal instruction is already difficult in underserved communities due to systemic issues, it was even more so at the time of data collection. For this reason, we employed general measures of resilience and LoC and used age and enrollment in music programs as a proxy for musical experience. We did not consider the years of music study of our participants, nor their motivations and perceptions of music programs. Furthermore, while adolescent musicians in this study came from the same instrumental music program, adult musicians had often participated in more than one program in their formative years, with a handful also teaching/facilitating learning in these spaces. Community music programs are not a “Blackbox,” but vary considerably in terms of curriculum, pedagogies, musical offerings, performance opportunities, and preparation of teachers and mentors. Unsurprisingly, the nature of music programs is known to have an impact on student learning and motivation. Future studies should not only describe these programs in detail but also find ways to quantify student musical participation in and out of programs, and beyond attendance. By doing so, they may clarify pedagogies and approaches that may support or hinder the development of musical and extramusical skills (Figure 1). It is also important that forthcoming studies examine resilience and LoC in students who dropped out of music programs, controlling for age, attendance (if available), and the nature and quality of such programs.

As noted, both resilience and LoC are susceptible to maturation and development (Nowicki et al., 2018; Livingston et al., 2025). For this reason, future investigations could adopt longitudinal and mixed methods designs with observational tools and measurements of resilience, LoC, and their building blocks. We also recommend that future research include a more controlled and larger sample and adopt gender as a variable of interest. Women and LGBTQ+IA populations still experience marginalization in some community music programs (Ilari & Bortz, contracted; Pimentel-Aguilar & Ramírez-González, 2024). Women from underserved communities have also been shown to drop out more frequently from community music programs in Latin America due to patriarchal structures, including the need to engage in domestic work from early on (Pimentel-Aguilar & Ramírez-González, 2024). In sum, there is an urgent need to delve deeper into gender issues in relation to musical participation, LoC, and the complex construct of psychological resilience. Such studies have clear implications for educational practice and, for issues of gender representation, equality, and societal well-being.

We also encourage future studies to revisit the work on resilience strategies outlined by Libório and Ungar (2009), particularly their notion of protagonism, which may assist in the dismantling of deficit-based frameworks associated with learners from the margins (Livingston et al., 2025). Our interviews (Ilari & Bortz, contracted) offered clues into protagonism as a strategy for resilience building. This included narratives of musicians leading and organizing youth in their local communities, teaching to “give back” to their programs and communities, and displaying what Brazilian educator Paulo Freire termed “stages of consciousness” (see Darder, 2014). Whether protagonism is exclusively a Brazilian phenomenon, and, more so, whether protagonism as a resilience strategy can be cultivated in and through community music programs, in Brazil and elsewhere, needs to be further investigated.

## 5. Conclusions

We conclude by stressing our hope that further research can expand from our modest findings and proposed Theory of Change (Figure 1), leading us towards a better understanding of musical participation, resilience and LoC. While LoC has been studied to a lesser extent in musical contexts, resilience is a construct that is commonly connected to community music programs and to the experiences of musicians from the margins. This is especially true for those who have succeeded despite years of immersion in chronic adversity. Solidifying this construct, identifying its building blocks (along with corresponding sources of support and hindrance), and understanding their associations with formal music instruction programs may help us better understand human experiences. Getting a better grasp of resilience and locus of control is also important for studies on well-being, in, through and with music. As our ToC (Figure 1) suggests, some of the building blocks of resilience and internal locus of control (e.g., positive emotions, confidence, competence, and sense of belonging) converge with the building blocks of positive functioning and well-being (Lerner et al., 2009; Seligman, 2011). Future research could help us tease out these components and ultimately design musical programs that serve multiple purposes, including the development of musical skills and the nurturing of non-musical capacities that help individuals thrive.

## Figures and Tables

**Table 1:** Six socioeconomic classes from the ABEP questionnaire.

A	B1	B2	C1	C2	DE
45–100	38–44	29–37	23–28	17–22	0–16

**Table 2:** Descriptive statistics: age, ABEP, RISC, LoC, and standard deviation.

	Minimum	Maximum	Mean	SD	Missing Data
Age (*n* = 102)	13	52	21.89	8.79	13 (11.3%)
RISC (*n* = 103)	41	125	88.91	16.85	12 (10.4%)
Musicians	45	116	91.98	16.34	
Untrained	41	125	86.59	17.69	
Adults	45	116	94.34	13.56	
Adolescents	41	125	84.69	18.76	
LoC (*n* = 99)	9	55	30.30	9.87	16 (13.9%)
Musicians	9	50	29.59	8.87	
Untrained	10	55	32.46	11.17	
Adults	10	55	30.88	9.07	
Adolescents	9	54	30.31	10.72	
ABEP (*n* = 96)	6	70	31.29	10.81	19 (16.5%)

**Table 3:** Model 1: unconditional regression model of RISC and LoC on music participation, age, and SES.

Outcomes	Predictors	Estimate	S.E	Est./S.E.	*p*-Value
RISC ON	Music Participation	0.073	0.110	0.668	0.504
	Age	0.232	0.089	2.598	0.009
	SES (ABEP)	0.083	0.093	0.894	0.371
LoC ON	Music Participation	−0.162	0.108	−1.503	0.133
	Age	0.064	0.091	0.706	0.480
	SES (ABEP)	−0.067	0.110	−0.608	0.543

**Table 4:** Model 2: conditional regression model of RISC and LoC on music participation, age, and SES.

Outcomes	Predictors	Estimate	S.E	Est./S.E.	*p*-Value
RISC ON	Music Participation	−0.359	0.132	−2.712	0.007
	Age	−0.071	0.103	−0.684	0.494
	SES (ABEP)	0.079	0.078	1.016	0.310
	Interaction	0.569	0.107	5.327	<0.001
LoC ON	Music Participation	0.307	0.116	2.640	0.008
	Age	0.297	0.065	4.611	<0.001
	SES (ABEP)	−0.059	0.078	−0.763	0.445
	Interaction	−0.576	0.081	−7.158	<0.001

## Data Availability

The data that support the findings of this study are available from the authors upon reasonable request.

## Abbreviations

The following abbreviations are used in this manuscript:

LoC	Locus of Control
SES	Social and Economic Status
ToC	Theory of Change
